# Supplementary material for: Integrated transcriptomic and methylome analysis reveals retinoic acid pathway activation after decitabine treatment in EBV-associated gastric cancer
Source: mBio. 2026 Feb 10;17(3):e03148-25. doi: 10.1128/mbio.03148-25 (PMC12977572; doi:10.1128/mbio.03148-25)
Supplement: Legend — Table S1 legend. [file mbio.03148-25-s0001.docx]

**Supplemental Table 1**. List of differential and common genes displayed in the upset plot in Figure 1E.
